# Pamphlets on the Cholera

**Published:** 1832-04-01

**Authors:** 


### Cholera Pamphlets.

### I. Letters on Cholera. By *Whitelaw Ainslie*, M.D.

The author of these Letters, so long and so favourably known to the public by his writings, appears to have been spending his time, *in otio cum dignitate*, in the country, after a long and active life in tropical climes, when the advent of an old enemy roused him from his quietude, and induced him to come forward once more on the public stage, with the hope of contributing his mite to that professional purse, which is not very full of information respecting the subject of Dr. A.'s inquiries. However unsuccessful, no one can say that the attempt is not laudable. Dr. A. does not step forward with a nostrum, a puff, a disinfecting agent, or an air-bath, for the purpose of filling his own purse. He can have no sinister or selfish object in view. Whatever may be the result, he is sure to be the loser; for, fortunately or unfortunately, books on cholera do not now pay the expence of the rough and coarse paper employed in working off the proof-sheets!

Our readers are aware that Dr. A. published a pamphlet, some time ago, broaching the doctrine, that an acid in the primæ viæ was the chief cause of the phenomena in cholera morbus, and proposing magnesia as a remedy. It was probably during the time Dr. A's work was passing through the press, that the discovery was made, by Drs. Clanny and O'Shaughnessy, that the contents of the stomach and bowels were alkaline in cholera, and, consequently, the proposed remedy useless! Commenta delet dies opinionum. We do not, indeed, attach much importance to the chemical discoveries, respecting either the blood or the secretions, in cholera. The changes induced in these fluids are secondary and very subordinate considerations. We know that, in dysentery, a large quantity of mucus is formed in, and daily discharged from, the bowels. Who would think that any medicine, acting on this mucus, would be of any avail in dysentery? Or can we cure dyspepsia, by neutralizing the acid perpetually generated in the first passages?

Dr. A. is of opinion, that the cholera of the present day is not a nova pestis. The Chinese have been acquainted with it for ages; and so have the Hindoos, who, in their *sastrums*, term it SWETA-RASA, or white fluid, from the appearance of the discharges. Dr. A. saw the disease, in 1815, with all the symptoms which it now presents, and, consequently, before its supposed origin, in Jessore.

"This is no place (says Dr. A.) to enter at large upon the causes of epidemics. These have puzzled the wise ones of all nations, from the period when men first began to reason, and differ on such points; and differ they will continue to do, till our *pneumatic* chemistry has made greater advances than it has yet done. The peculiar nature of the morbific miasmata giving birth to distempers, must of course be essentially different, corresponding with each distinct disease engendered; whether *bred* and *brewed*, to speak figuratively, on the noisome face of a dark morass,—within the confines and narrow lanes of a large, crowded, ill-supplied, and ill-ventilated city—on the ensanguined plain, amidst thousands of unburied dead—or in the obscurity and damp of some vast wood, where animals breathe and plants grow, which never felt the purifying influence of the sun's rays—or within the confines of the prison-house, where sorrow and remorse render still more deleterious the vapours of the dungeon." 5.

When president of a medical committee, appointed to enquire into the epidemic fever, which devastated India in 1809-10 and 11, Dr. A. spared no pains to investigate its causes; but in vain. He is disposed to attribute that and the present epidemic to some peculiarity in the electric fluid, not yet ascertained. A good many are now inclined to the same opinion. With respect to the question of contagion, Dr. A. is more inclined to the con than the pro; but is not decided either way.

### II. Cholera, its Character, Treatment, Identity, &c. By *Dr. Thackrah*, of Leeds.

This gentleman is already favourably known to the profession by his writings, and especially by his Essay on the Blood. It appears that Dr. Thackrah, like several other zealous members of the profession, left his home and private practice, in order to observe, on the spot, the nature, propagation, and treatment of cholera morbus. The present pamphlet contains the result of his observation. We shall, doubtless, have many other productions of the same kind, from those who were spectators of the epidemic on its first visitation to our shores. Dr. T. divides cholera into three forms—the mild autumnal bilious flux—the congestive or purple cholera—and the cholera with marked defect of the secretions.

Among other preliminary or premonitory symptoms, Dr. T. remarked diarrhœa. We have often suspected the truth of that general remark, that the sensorial functions remain unaffected till the last in cholera. Dr. T. admits that this is sometimes the case, but by no means generally so.

"The integrity of the mental faculties has been stated as a peculiar symptom. Indeed, it is sometimes very remarkable. The mind seems to sit unimpaired and serene, amidst the ruins of organic life. But the phenomenon is by no means universal, nor even, I think, general. Consciousness, as far as I have observed, is generally reduced; there is occasionally the pettishness of a child half wakened, stupor, sometimes difficulty of comprehension, and sometimes even coma." 5.

Vomiting and purging are often absent in the worst cases, though the diarrhœa may have preceded the severe attack. Sometimes the patient craves for food and drink. The ejected matters are, at first, the contents of the stomach, often tinged with green bile—afterwards, the pale fluids characteristic of cholera.

"Modified by constitution and circumstances, cholera presents great diversities of character and combination. In the present epidemic we may readily find every variety, from the mildest diarrhœa, or bilious flux, to the purple disease. Such is the case now at Newcastle and Gateshead. The attacks at first were chiefly of the most severe kind; but the disorder at present is comparatively mild, and of a character familiar to English practitioners. On its invading a place, epidemic cholera is generally violent and destructive; but, in a short period, it seems to sink into ordinary disease;—to revive, however, with all its malignity, at the next town or district.

*Dr. Daun* informed us, and the remark has been made by others, that in India, when a patient recovered from the direct effect of cholera, no danger was apprehended, and he regularly advanced to health, but that at Sunderland he saw a new and alarming train of symptoms frequently supervene and destroy in a few days. These were witnessed on the continent of Europe, and are fully described in the reports of *Drs. Russell* and *Barry*. They constitute the consecutive fever. A patient rallying from the congestive stage of cholera is found to have the skin hotter than natural, the pulse hard and wiry, the tongue furred, drier than before, and red at the edges, and to complain of headache. The urine is high-coloured; there is tenderness on pressure of the abdomen; soon the tongue becomes quite brown, and dry: the head is more affected; in a word, a form of typhus destroys the patient in a few days. We visited the cholera hospital at Sunderland when the inmates were said to exhibit chiefly the consecutive fever; but to our observation they seemed to labour generally under mild congestion, rather than typhus. The pulse was rarely above 100; the skin was not hot and parched, nor the tongue dry or brown." 7.

The consecutive fever he conceives to depend on gastro-enterite or cephalitis.

Of the primordial germ of cholera we know nothing, and of the mode of its propagation, little. Much has been written on the spread of the disease. The medical press teems with disputes on the contagious or non-contagious—on the infectious or non-infectious—on the communicable or incommunicable character of the malady. But have the disputants accurately examined the atmosphere by which, or through which, the disease is propagated? The chemists find little difference between the pure air of the mountains, and that of confined apartments fouled by the respiration of a crowd; and on marsh miasms or malaria, little light has yet been thrown. Till this subject be developed, nothing fully satisfactory can be established, on the subject of infection, the character of endemics, or the spread of epidemics. We may infer, however, from the information hitherto adduced, that peculiar states of the atmosphere, connected with its electricity, or with terrestrial exhalations, produce a disease, which, in proportion to the number of patients, and the unfavourable circumstances in which these patients are placed, becomes aggravated in character, assumes an infectious nature, and is communicated from person to person. This communication, however, is not uniform. Some states of atmosphere promote it, some resist it. Neither does the disease attack persons indifferently. A great proportion of every population is invulnerable; cholera strikes down the predisposed; and when these have been attacked, the disease disappears, for a time at least, in the town or district." 24.

Dr. Thackrah describes an epidemic cholera which prevailed in the town and neighbourhood of Leeds, in the year 1825. It was preceded by an unusually wet Spring, with easterly winds, while the Summer was remarkably hot and sultry. The cholera of August, September, and October, presented almost all the symptoms of the present epidemic. It was more generally diffused, but not so fatal. In Leeds, 43 per cent. of the inhabitants were affected by the cholera. At Moor Allerton, the ratio of mortality was about 4 per cent. of the sick. The following passage may inspire the faint-hearted cockney with hope.

"1st. Cholera was much more prevalent in the country than in the town. The atmosphere, the great agent, of course, in all epidemics, pure in the country, and fully subject to all the changes of season, is much more artificial in large and populous towns. Smoke, animal effluvia, and probably also diversities in the electrical state of the air, greatly diminish its susceptibility to those changes, morbid as well as healthy, which nature effects in successive years. Thus, while the impurity of atmosphere generally prevents townsmen enjoying robust and buoyant health, it also shields them from the violence of epidemics. Typhus frequently affords an illustration. Prevalent and fatal in the fine and elevated village of Rawden, marked neither by poverty nor filth, it is rarely severe in the sheltered town of Leeds, which of course abounds with the usual fomites of disease.

2nd. Lofty situations, and those especially which are almost destitute of wood, were more subject to cholera, than plains and vallies.

3rd. Cholera seized chiefly the poor and debilitated. In several pauper families at Moor Allerton, we found that every individual had suffered, more or less, from the epidemic, and that the three who died in the district examined were debilitated females, destitute of the comforts, and almost of the necessaries of life: while the fatal case we attended, not far distant, was a man oppressed with a large family, ill fed, and hard worked. The comparative exemption of the upper classes of society, I have often observed in my own practice in the town ; and in the country this fact is more remarkably exemplified. At Moor Allerton, in the district examined, there are six residences of merchants and gentlemen, containing 50 individuals, and of those only one had cholera—2 per cent. As a contrast, we found in six of the poor houses, containing 39 inhabitants, 21 had been affected with the disease—53 per cent. A similar observation was made at Kirkstall. The power, in fact, of most agents of disease is diminished in the inverse proportion to the animal vigour of the individuals exposed.

These observations, written in 1825, seem to refer cholera to atmospheric influence, independent of infection or contagion. But a note, made at the same time, shews that there was also another mode of propagation suspected. 'Several observations lead me to suppose that cholera is sometimes infectious. I do not assert the fact, but I have observed as many circumstances concur to favour this opinion in reference to cholera, as in fever—at least in the cases I have seen in this neighbourhood for the last nine years.' *A recent examination of the details of the epidemic produces the conviction that, although it originated in a wide-spread constitution of atmosphere, it was capable, in certain circumstances, of being communicated from person to person.*" 30.

From the latter part of the foregoing extract, it is evident that Dr. Thackrah is a contingent contagionist. Our intelligent author dedicates a section to the identity of English and Indian cholera." He observes that the "bulk of the profession, and the principal writers on the disease, have supposed a wide difference between the epidemic cholera of India and our own." But from the perusal of written descriptions, and from personal comparison of what he saw in his own practice formerly, and now at Newcastle, he comes to a very different conclusion. Dr. T. details ten cases of the Leeds' epidemic, of 1825, and no one can peruse these cases, without acknowledging that it is impossible to separate them from what is called the Asiatic cholera, as far as the phenomena of the disease can be taken as criteria. We are tempted to quote two or three cases.

"In August, 1824, a strong man was attacked with vomiting and purging. The evacuations were quite devoid of bile. The cramps exceeded in violence any thing I had before seen. Such were their convulsive character, that he was once thrown out of bed, and three men were afterwards required to hold him. The agony made him cry out with vehemence. This patient also recovered.

In September, 1825, a debilitated and ailing female, attended by *Mr. Corsellis*, was seized at 3, a.m. with vomiting and purging. Most distressing cramps speedily ensued ; the surface became cold ; the countenance sunk, and, to use the phrase of a woman who attended her, *was* '*all blue as violet.*' *The stools were colourless.* She could not retain them. She died about eight in the evening, 17 hours from the commencement of the urgent symptoms. On laying out the body, the women particularly remarked its blue, black, mottled appearance. *One leg remained flexed by spasm, its foot resting on the shin of the other.*

An elderly woman, who attended the funeral of the preceding, was attacked a week after. After shearing corn all day, she went to bed well at night, but at eight a.m. was seized with vomiting and purging, frequent and profuse. The countenance was much shrunk ; the eyes sunk, and surrounded with a blue circle ; the whole surface cold, and extremities quite pale. The evacuations were not seen by her medical attendant, but they were said to be yellow and offensive. She had no cramps ; she lay indifferent to external objects, and constantly dozing. Some reaction took place next day ; but on the third morning she became worse, continually complained of cold, and sunk at night.

These two females lived in a hamlet, the houses of which are as close to each other as those of a town. After these deaths, cholera spread among the inhabitants, and left not a house unattacked.

My excellent friend and quondam pupil, *Dr. Whitehead*, of Beverley, has referred to me a case which occurred while he was with me, in the year 1825, and of which I regret that I cannot find any details recorded. A man was attacked with violent symptoms of cholera, at two o'clock in the morning, and sunk at ten—eight hours, consequently, from the invasion of the disease. On post-mortem examination, our principal remark was the large quantity of *albuminous matter* in the small intestines. The stomach was greatly contracted.

In September, 1825, a labouring man, 63 years of age, was affected with diarrhœa for a few days, but so mild as not to interfere with his usual avocations. In the night, he was seized with vomiting, and when seen three hours after the attack, by *Mr. Scholefield*, then my assistant, he had the facies Hippocratica, extremities cold, and radial artery without pulsation. He died in two or three hours." 36.

We ask the advocates of *imported cholera* to read the foregoing cases without prejudice. But the appeal is vain. They cannot see any thing but through the yellow medium of quarantine creeds!

"The cases which I have adduced are not offered as fair samples of English epidemics. They are rare; while milder forms of the disease are abundant. They show not what English cholera generally is, but what it can be—the appalling form which it sometimes presents. This aggravated character, we can only, in the present state of our knowledge, ascribe to peculiar and obscure modifications of atmosphere, to predisposition, or circumstance. In what does it differ from the Malignant Cholera, the Spasmodic Cholera of India? I believe in none. I do not, of course, mean to assert that the cases to which I refer, are accordant in every particular either with those in India, or with each other. No two cases can in any country, or of any disease, be expected to have precisely the same symptoms. But I contend that the signs which are considered to characterize the Indian Cholera are found in a marked and decided degree in the cases just stated. Thus we have the peculiar character of the evacuations —the sudden and great prostration of strength—the extraordinary reduction of pulse—the shrunk and purple countenance—the loss of voice—the purple, or pale, contracted state of the extremities—and death sometimes in a few hours. I conceive, therefore, that no fair reasoner can refuse to admit the identity of the disease. A physician sent down by Government to ascertain the character of the Cholera in the north of England, has repeatedly declared that he could distinguish the Indian from the English *only* by the *prevalence* of the former—the *number*, not *nature*, of the cases giving the distinctive character of the Indian! Cholera in England has certainly not produced as great a mortality as Cholera in India. Neither has inflammation of the liver; yet no one considers Indian Hepatitis as distinct, in nature, from English. The character of the people, their habits and food, climate, and other circumstances, have produced a mortality from both diseases which seems enormous to the English practitioner. Could an Indian army, however, with its train of followers—enfeebled, debauched, and fatigued, deficient in protection from the weather, deficient, especially, in nourishing food—have been encamped in England in 1825, we should have beheld, I conceive, the most appalling form of the disease. The concentration of individual cases in bad circumstances, would have produced a poison as communicable from person to person, and as fatal as the present dreaded epidemic. Had the English and Indian Cholera been attentively compared, medical boards would not have been perplexed in their attempts to declare the symptoms which distinguish the one from the other, nor have considered an absence of bile the peculiar and distinguishing character of the Indian malady. But men, not familiar with the epidemics of former years, have determined the character of the present. The Indian practitioner has been sent to decide between two diseases, when he was intimately acquainted with but one. Had the identity of character in the epidemics been ascertained by the medical men, who have given opposite names to the disease in particular places, official papers would have been more satisfactory and accordant; the distressing doubt of the public would have been prevented; and more attention would have been paid to the important and practical parts of the subject." 40.

The treatment of cholera need not detain us long. Dr. T. considers venesection as particularly indicated—first, as reducing the turgescence of the internal vessels—secondly, as rendering the blood more fluid, by promoting absorption from the serous cavities—thirdly, as reducing spasm—fourthly, as promoting the natural secretions. Dr. T. has witnessed decided benefit from this practice in Sunderland during the present epidemic, and also in his own practice, during the Leeds epidemic of 1825. He has also observed the most beneficial effects from scruple doses of calomel. On the other remedies we need not dwell. We have been much pleased with the candour, good sense, and professional talent displayed in this pamphlet.

